# “How Did We Get Here?”: Topic Drift in Online Health Discussions

**DOI:** 10.2196/jmir.6297

**Published:** 2016-11-02

**Authors:** Albert Park, Andrea L Hartzler, Jina Huh, Gary Hsieh, David W McDonald, Wanda Pratt

**Affiliations:** ^1^ Department of Biomedical Informatics School of Medicine University of Utah Salt Lake City, UT United States; ^2^ Group Health Research Institute Seattle, WA United States; ^3^ Department of Biomedical Informatics School of Medicine University of California San Diego La Jolla, CA United States; ^4^ Department of Human Centered Design & Engineering University of Washington Seattle, WA United States; ^5^ Information School University of Washington Seattle, WA United States

**Keywords:** consumer health, health informatics, health care information systems, similarity measures, Internet communications tools, Web-based interaction, collaborative and social computing systems and tools

## Abstract

**Background:**

Patients increasingly use online health communities to exchange health information and peer support. During the progression of health discussions, a change of topic—topic drift—can occur. Topic drift is a frequent phenomenon linked to incoherence and frustration in online communities and other forms of computer-mediated communication. For sensitive topics, such as health, such drift could have life-altering repercussions, yet topic drift has not been studied in these contexts.

**Objective:**

Our goals were to understand topic drift in online health communities and then to develop and evaluate an automated approach to detect both topic drift and efforts of community members to counteract such drift.

**Methods:**

We manually analyzed 721 posts from 184 threads from 7 online health communities within WebMD to understand topic drift, members’ reaction towards topic drift, and their efforts to counteract topic drift. Then, we developed an automated approach to detect topic drift and counteraction efforts. We detected topic drift by calculating cosine similarity between 229,156 posts from 37,805 threads and measuring change of cosine similarity scores from the threads’ first posts to their sequential posts. Using a similar approach, we detected counteractions to topic drift in threads by focusing on the irregular increase of similarity scores compared to the previous post in threads. Finally, we evaluated the performance of our automated approaches to detect topic drift and counteracting efforts by using a manually developed gold standard.

**Results:**

Our qualitative analyses revealed that in threads of online health communities, topics change gradually, but usually stay within the global frame of topics for the specific community. Members showed frustration when topic drift occurred in the middle of threads but reacted positively to off-topic stories shared as separate threads. Although all types of members helped to counteract topic drift, original posters provided the most effort to keep threads on topic. Cosine similarity scores show promise for automatically detecting topical changes in online health discussions. In our manual evaluation, we achieved an F1 score of .71 and .73 for detecting topic drift and counteracting efforts to stay on topic, respectively.

**Conclusions:**

Our analyses expand our understanding of topic drift in a health context and highlight practical implications, such as promoting off-topic discussions as a function of building rapport in online health communities. Furthermore, the quantitative findings suggest that an automated tool could help detect topic drift, support counteraction efforts to bring the conversation back on topic, and improve communication in these important communities. Findings from this study have the potential to reduce topic drift and improve online health community members’ experience of computer-mediated communication. Improved communication could enhance the personal health management of members who seek essential information and support during times of difficulty.

## Introduction

To illustrate the importance of addressing topic drift in online health communities, consider the case of Anne who was curious about a side effect she was experiencing with a newly prescribed medication for attention deficit hyperactivity disorder (ADHD). She was worried that the side effect would get worse and wanted to hear about other people’s experiences. She started a discussion regarding the drug and side effects in an online discussion group. Other online community members joined the discussion and shared their experiences as ADHD patients. When someone mentioned taking medication to prevent being fired from work, the topic of conversation changed to ADHD and work performance, including a discussion of the Americans with Disabilities Act (ADA) and legal advice. Ultimately, the conversation ended when one member repeatedly posted about his negative experience obtaining ADA assistance. Anne’s specific question regarding her medication side effect was never answered, and she decided to stop taking the drug. If she had learned how others dealt with the side effect and that it did not get worse, she might have continued the treatment. What could Anne, moderators, or the online community have done to get Anne’s questions answered?

Anne experienced topic drift [1], where the focus of conversation changes as a discussion progresses. In a conversation, topics naturally and continuously change [2]. However, topic drift occurs frequently in computer-mediated communication (CMC) and can be a source of incoherence [3] and frustration. Moreover, topic drift can hinder meaningful social interaction [4] and knowledge construction [4,5]. Despite the importance of maintaining the goal (eg, acquiring information or support on the initiating topic) and topic of discussion, drift can still occur. For example, in a previous study of social-oriented chat on the Internet, nearly half (47%) of conversation was considered off-topic [6]. Additionally, keeping conversation on topic has been shown to be difficult even for highly focused discussion groups, such as those that discuss the Oklahoma City bombing [7] or health and fitness [8].

Previous studies on topic drift have focused on different domains and CMC methods, including email-based newsgroups [7], online discussion about open source software design via mailing lists [9], chats about classical music [10], and pharmacy class meeting chats [11]. Although for some domains, topic drift can be inconsequential or even a natural course of conversation, for other sensitive domains, such as health, topic drift can pose serious consequences—as Anne’s case demonstrates. Online health communities allow patients to cope and manage their illnesses through social interactions while providing means to overcome barriers, such as geographical isolation or stigma from certain diseases. Previous studies have shown a correlation between participating in online health communities and improvement of depression [12-16], anxiety [14,16,17], stress [14,15], negative mood [18], and health outcomes [19,20]. Although topic drift can hinder obtaining these benefits, in-depth analyses of topic drift in health discussions have yet to be reported, and thus it is not well understood.

Analyzing topic drift can shed light on the overall community experience. For example, Lambiase found that emotionally aggressive postings led discussion away from the original topic and led participants to unsubscribe or remain inactive [7]. Similarly, Selfe and Meyer found that participants who used powerful and persistent language controlled the topic of conversation while limiting the opinions of others [21]. Few online communities employ moderators to govern discussion and create an engaging and respectful community culture [22]. In a moderated community, it is reasonable to assume that moderators will provide a structure to keep topics relevant to the goal of the thread and community as well as counteract aggressive and persistent postings. Whether moderators or other members provide effort to counteract topic drift—returning back to the original goals and topics of the discussion—is an unanswered research question.

According to Hobbs, 3 conversational devices attributed to topic drift in dialogues are semantic parallelism, chained explanation, and metatalk [1]. Semantic parallelism occurs when a small portion of a topic gradually changes to other topics with similar and relevant properties. Chained explanation occurs when an explanation seems more interesting than the current topic and becomes the new topic. Metatalk occurs when participants evaluate the drifted topic and change it back to the original topic of conversation. The first 2 devices are cases of gradual topic drift, whereas metatalk opposes the drift by explicitly encouraging a return to the main topic.

Many manual analyses of topic drift [3,7,11] have used Hobbs’ theoretical conversational devices of topic drift to explain how topics change in both synchronous (eg, chat) [3,11] and asynchronous (eg, email, forums) [7] CMC. Other topic drift studies that did not employ Hobbs’ theory also manually assessed topic drift [9,10]. One limitation of manual assessment is the inherently subjective nature of determining topic drift [11]. Moreover, such analyses require tremendous effort and time.

Returning to our motivating scenario, people like Anne openly discuss and seek information and support in online health communities, such as WebMD [23,24]. These online health communities provide psychosocial benefits (eg, adaptive coping) [25] as well as useful health information [26,27]. Although topic drift can hinder these benefits, the effects of and members’ reactions toward topic drift in online health communities have not been studied. Despite the importance of staying on topic, counteracting efforts to topic drift have received limited attention. *Who* provides this counteracting effort to topic drift in topically focused communities is unknown. Furthermore, automated techniques have the potential to detect both topic drift and counteracting efforts but are unexplored in online health communities. Answering these open issues is important to inform support that helps valuable online health communities thrive.

## Methods

### Data

The data for this study consist of posts from moderated, disease-specific WebMD communities. WebMD is one of the most popular health information sources for health consumers [28], thus we examined posts from WebMD communities. We selected specific communities that vary with respect to disease and illness characteristics to cover wide aspects of health (ie, biological, psychological, and sociological) and representative demographics (ie, age and gender). WebMD communities also employ staff moderators and medical doctors (MDs) who have clearly defined community roles compared with regular members (ie, “users”), which allowed us to analyze the relationship between community member role and both topic drift and counteraction to topic drift. To understand how staff moderators and MDs influence topic drift, we considered the total number of available staff moderators and MDs as well as their total number of posts in community selection.

We selected 7 WebMD communities: (1) attention deficit hyperactivity disorder (ADHD), (2) breast cancer, (3) diabetes, (4) heart disease, (5) multiple sclerosis (MS), (6) pain management, and (7) sexual health (Table 1). We downloaded all publicly available posts from these seven communities. Then, we removed threads without posts replying to the initial post. Communities averaged between 2.86 and 7.78 posts per thread, and across all communities the average thread length (TL) was 6.76 posts.

The University of Washington Institutional Review Board determined this study exempt from review.

### Research Questions and Topic Drift Analysis

To understand topic drift in online health communities, we characterized the severity of topic drift as either *gradual* or *abrupt topic drift*, determined by the degree of topical change from the previous post to the current post in a thread (RQ1). Gradual topic drift refers to small degrees of topical change in which the current topic is related to the previous topic. We considered a complete change of topic as well as topic domination as abrupt topic drift. Topic domination was measured through previously identified tactics—using a high volume of messages [29] and ignoring conventional conversational rules [30] (eg, disrupting the conversation or ignoring the main goals of the thread).

**Table 1:** Characteristics of 7 WebMD communities studied. ADHD: attention deficit hyperactivity disorder; MS: Multiple Sclerosis.

	ADHD	Breast cancer	Diabetes	Heart disease	MS	Pain management	Sexual health
Dates data were collected	7/2005 to 6/2012	8/2007 to 5/2012	6/2007 to 5/2012	3/2008 to 5/2012	11/2007 to 1/2013	9/2007 to 6/2012	9/2007 to 1/2013
Posts, n	8704	21,612	64,085	11,874	27,412	27,333	68,136
Threads, n	2313	3227	8242	4146	4943	4656	10,278
MDs and staff, n	13	13	16	10	11	12	11
Users, n	2984	2147	4385	3815	2710	5843	13,624
Power users, n	5	17	36	8	16	17	32
Mean thread length (TL)	3.76	6.70	7.78	2.86	5.55	5.87	6.63
Median TL	3	5	5	2	4	4	4
Max TL	85	88	97	71	68	97	99

**Table 2:** WebMD datasets used to answer research questions.

		Dataset analyzed	Gold standard used for evaluation
Qualitative and systematic analyses	RQ1	416 posts from 50 randomly selected threads with minimum of 6 posts	N/A
	RQ2	185 posts from 168 unique threads selected with key words: “hijack” and “off topic”	N/A
	RQ3a	50 randomly selected threads from RQ1 and an additional 20 purposively selected threads with 187 posts	N/A
Quantitative analyses	RQ3b	229,156 posts from 37,805 threads	N/A
	RQ4	229,156 posts from 37,805 threads	77 posts were assigned using key words and 70 posts were manually evaluated to create a gold standard
	RQ5	229,156 posts from 37,805 threads	50 randomly selected posts from 50 threads were manually evaluated to create a gold standard

We also categorized the types of topic drift as either *global* or *local topic drift*, based on the characteristics of topic drift [7,31]. Local topic drift refers to initiating a new topic unrelated to the current topic of conversation (ie, when someone brings up a new topic within a thread that does not relate to the original post but stays within respective communities’ goals). In contrast, global topic drift refers to discussions outside of the respective communities’ goals (ie, when someone starts a new thread that does not relate to the focal topic for that community).

To understand topic drift in a health context, we manually analyzed topic drift in online health communities to answer 3 initial research questions:

RQ1: How does local topic drift occur in threads?

RQ2: What are members’ reactions and meta-discussions towards topic drift in explicitly identified topic drift threads?

RQ3: Who brings the topic back to the original topic of threads (ie, counteraction effort)?

Based on results from RQ1-RQ3, we developed an automated approach to identify both topic drift and efforts by members to prevent or counteract such drift. Many of the studies on topic drift manually analyzed conversations [3,7,9-11]. The manual method is accurate but is labor intensive and limited to small datasets. However, in the field of information retrieval, researchers have long used automated methods to cluster similar topics [32] as well as to detect and track topic changes on various streams of text from newswire, television, radio, and Web broadcast news shows [33]. One of the more widely used methods is similarity measurement of terms in text segments using thresholds based on term frequency-inverse document frequency (tf-idf)—a statistical representation of importance of a word to a document in a collection of documents [34]. Likewise, we applied a cosine-similarity metric and vector space model to assess similarity between posts within the same thread to detect both gradual and abrupt local topic drift.

We chose to use cosine similarity because it is one of the most widely used and thoroughly studied measures [35]. One advantage of cosine similarity for analyzing various types of consumer-generated text is that the measurement normalizes the text length during the comparison. Thus, longer replies would not necessarily be considered to have a higher number of shared words and appear to be more on topic. To measure topic drift by cosine similarity between posts, we first represented each post as a vector in N-dimensional vector space, where N is the number of unique terms across all posts and the value is the frequency with which terms occur in that post. Cosine similarity measures the cosine of the angle between 2 vectors representing the posts. The resulting cosine similarity score ranges from 0 to 1. A score of 0 indicates no shared terms between the 2 posts, whereas a score of 1 indicates all terms and the relative proportion of the terms used are exactly equal. We calculated tf-idf at the community level to reflect important terms discussed across each of the 7 communities. Then, we automatically measured the general trend of topic drift in the 7 online health communities.

To examine the application of our automated approach, we answered the following research questions:

RQ4: How accurately can local topic drift be detected automatically?

RQ5: How accurately can counteraction effort be detected automatically?

In the following section, we present a summary table of datasets that we used and delineate methods for each research question. Table 2 overviews the WebMD datasets used to answer each research question, including gold standards used for evaluations.

### RQ1. How Does Local Topic Drift Occur in Threads?

We first manually analyzed 50 randomly selected threads with at least 6 posts, which was the average number of posts in all 7 WebMD communities and provided enough posts per thread to perform an in-depth, manual analysis of topic drift. The heart disease community was randomly selected to have 8 threads, whereas the other communities had 7 threads to make up our randomly selected 50 threads for this analysis. We systematically identified a number of main topics in each of the posts and examined whether and how many of those main topics changed as threads evolved. Using this information, we categorized topical changes into gradual (ie, at least one previous topic remained) or abrupt topic drift (ie, no previous topic remained). We also qualitatively analyzed and identified possible sources and the general trend of topic drift following an open coding process [36].

### RQ2. What Are Members’ Reactions and Meta-Discussions on Topic Drift in Explicitly Identified Topic Drift Threads?

We began this analysis using self-identified topic drifted threads. We analyzed member-identified threads where topic drift occurred. We analyzed reactions and meta-discussions when topic drift was apparent to members to understand the consequences of topic drift in overall community experience. Terms such as “anyway,” “speaking of X” [37], “so,” and “oh” [38,39] were identified as markers that initiate topic drift in a face-to-face conversation. Similarly, we used a lexicon-based extraction approach to extract threads containing explicit expressions of topic drift with the variations of the key terms “hijack” or “off topic,” which are known markers for local and global topic drift in CMC. “Hijack” or “hijacking” a thread is a colloquial term in CMC denoting changes in topic from original posts [40] (ie, local topic drift). This term was also used and well understood in the communities we analyzed. “Off topic” is another term that was used to describe topics irrelevant to the main discussion in CMC [7,41]. “Off topic” can indicate either local or global topic drift. We extracted posts that contained either key terms in the body of the posts (ie, not the title). Then we manually referred back to the preceding posts and reviewed the context of the conversation to ensure that the key terms were used for rhetorical strategies to change topic, gain control over the topic, or indicate off-topic content in the post. In other words, the key terms had to be used to indicate or relate to either global or local topic drift. We then qualitatively analyzed these threads with respect to meta-discussion and members’ emotional reaction towards topic drift. We then applied a Mann-Whitney U test (U) [42] to statistically compare the length of off-topic threads to the rest of the threads to further investigate members’ usage of these off-topic threads. We applied nonparametric tests because our data were not normally distributed. Given the large sample of threads, we report effect sizes (*r*) using rank-biserial correlation [43].

### RQ3. Who Counteracts Topic Drift?

To understand who brought the topic back to the original topic of a thread, we examined counteraction in 2 phases using (1) manual and (2) automated methods. First, we manually analyzed threads to determine who counteracts (RQ3a). The concept of staying on topic is related to one of Hobbs’ conversational devices, metatalk [1]. Metatalk also can be about a discussion regarding their conversation. Therefore, for clarity, we did not use the term metatalk. Instead, we defined a community member’s effort to stay on topic as the *counteraction* to topic drift.

In addition to the 50 randomly selected threads in RQ1, we purposely sampled an additional 20 threads with at least 6 posts, in which members with defined roles (ie, MDs and staff moderators) participated to understand how these members impact or counteract topic drift. We chose a purposive sampling strategy because participation of members with defined roles was relatively limited, and we were not able to sample enough threads with their participation using random selection. For posts in each of the 70 threads, we de-identified the community member identification (ID) and manually examined the role of the community member who made the effort to counteract topic drift (ie, users vs MD or staff moderators).

In the manual analysis, first we systematically identified main topics in each of the posts and noted neglected topics in subsequent posts. Second, we looked for any rhetorical cues to previously neglected topics. For example, we observed statements like “to answer your question on” that were often used when counteracting topic drift. Third, we noted posts discussing previously neglected topics without any rhetorical cues for counteraction. Fourth, based on this information, we categorized each post as counteracting or not counteracting.

In the second phase (RQ3b), we automatically detected counteraction efforts and noted the role of the member who made that effort. According to Dorval, the topic of conversation is not static but a constantly changing feature [2]. Furthermore, Lambiase showed that the topic of conversation slowly drifted from the original topics as conversation progressed in CMC [7]. Assuming the same natural deviation happens in the online health discussions, we focused on the irregular increase of cosine similarity scores to detect counteractions to topic drift in threads. The irregular increase of similarity score could indicate that the current post contained more relevant topics to the initial post compared to the previous post (ie, threshold), a sign of a counteraction to topic drift.

As with our approach to detect topic drift, we applied the cosine-similarity metric and vector space model with tf-idf to detect counteraction efforts. We automatically measured who (ie, which type of member) made counteractions. To understand how people in defined roles provide counteractions, we categorized the members as moderators (ie, staff/MD) or users according to their community member identification (ID). We categorized users further as *original posters*, *power users*, and *regular users*, which were mutually exclusive roles for individual threads. We defined original posters as users who initiated a thread, power users as users who posted more than the average number of posts by moderators, and remaining users as regular users.

For each role, we estimated average counteraction effort. The unit of analysis was a role within a thread (ie, original posters, staff/MD moderator, power user, regular user). Even though a given member can play more than one role, for purposes of this analysis, we assumed that members’ counteracting behavior was independent if they played different roles in different threads. To estimate average counteraction effort, we counted the number of occurrences of counteraction each member made in each thread. Because the most active members have a greater chance of providing such effort, we normalized each member’s total counteraction occurrences divided by the total number of replying posts they made in the thread (ie, excludes the original post), thus converting the occurrences into percentages (ie, “counteraction effort”). We averaged the mean counteraction effort for each member when acting in the same role. Then we averaged the mean counteraction effort for each role.

To compare counteraction effort among roles, we applied a Kruskal-Wallis H-test (X^2^) [42]. We then conducted post-hoc pairwise comparisons of counteraction effort between roles using Mann-Whitney U tests (U) [42] with a Holm **-**Bonferroni correction to *P* values. Given the large sample of members, we report effect sizes (*r*) for the pairwise comparisons using rank-biserial correlation [43]. Finally, we compared results from automated measurement (RQ3b) with results from the manual measurement (RQ3a).

### RQ4. How Accurately Can Local Topic Drift Be Detected Automatically?

We evaluated our automated topic drift detection technique with self-identified topic drift and “on-topic” posts. First, we used posts from RQ2 that contained key terms: “hijack” or “off topic” as positive cases that our detection system should recognize as low in similarity measurement, given that members explicitly indicated the off-topic nature of the post. Because the interpretation of topic drift can be subjective [11], we relied on members’ explicit indication of topic drift as the gold standard for positive cases. To ensure quality, we manually examined and removed posts from this analysis if (1) the keyword hijack literally meant illegally seize or steal (a few posts were about the 9-11 tragedy), (2) the keywords were used to describe the definition of an acronym (eg, *“OT means [...]”*) or community nomenclature (eg, *“hijacking a thread means […]”*), (3) the keywords had a modifier to indicate lesser degree (eg, *“may be slightly off topic”*), (4) the keywords were used in meta-discussion about off-topic discussions, or (5) the keywords were used to start new off-topic threads (eg, *“OFF TOPIC BUT […]”*). These were stricter criteria than RQ2 because this also removed global topic drifts along with the posts that described OT and lesser degreed local topic drifts.

To identify negative cases, we first used posts from RQ2 if (1) the posts negated the keyword (eg, *“this is not off topic”*) or (2) community members had shown intention to bring topics back to the original post (eg, *“your question got hijacked, I’ll try to get it back on track”*). Because there were only a few negative cases, we added 70 manually selected “on-topic” posts with little or no topic drift that the detection system should recognize as high in topical similarity from the RQ3a qualitative analyses. We made these selections and adjustments prior to the evaluation process without any information on their similarity scores.

Using these positive and negative cases as a gold standard, we calculated the precision, recall, accuracy, and F1 score of the automated topic drift detection system compared to the average score of posts in the same position of all threads. The position of the post was important because we expected the topic of conversation to naturally change [2,7] and the cosine similarity scores to decrease accordingly as conversation progresses. Precision measures the proportion of predicted positive instances that are correct. Recall measures the proportion of positive instances that are predicted. Accuracy measures the percentages of correctly predicted instances among the total number of instances examined. F1 score is the weighted harmonic mean—reflecting both performance and balance—of precision and recall. In all measures, higher scores reflect better performance.

### RQ5. How Accurately Can Counteraction Effort Be Detected Automatically?

To evaluate our approach to automatically detect counteraction efforts, we used 50 new, randomly selected posts from 50 threads: 25 posts with a natural decrease in similarity score and 25 counteracting posts with an increase in similarity score. We de-identified the origin of the 50 posts then manually categorized as natural topic drift or counteraction to topic drift, while referring back to initiating and other previous posts to understand the context. Using manual assessment of 50 posts as a gold standard, we then calculated the precision, recall, accuracy, and F1 score of the automated topic drift detection system.

## Results

In this section, we present the results of 3 manual analyses (RQ1-RQ3a) and then the results of quantitative analyses (RQ3b-RQ5) for the 7 moderated online health communities.

### RQ1. How Does Local Topic Drift Occur in Threads?

We manually analyzed 416 posts from 50 threads. Our systematic analysis showed that in most threads, the topic changed gradually— *gradual topic drift* —in which topics remained in the discussion while few topics were newly introduced or neglected (ie, semantic parallelism). This gradual change occurred among posts in nearly every thread. However, threads generally (46/50, 92%) stayed within the global frame of community topics, including symptoms, treatments, side effects, insurance issues, and emotional support for the specific community. On average, threads started with 3.44 topics and 1.05 topics were carried from post to post, while 1.58 topics were newly introduced. The following are themes associated with the severity and sources of topic drift.

#### Severity and Sources of Topic Drift

Abrupt topic drift occurred in 22% (11/50) of manually examined threads. The following is an example thread (Example Thread 1) from the Heart Disease community that showed abrupt topic drift, in which Poster_C controlled and changed the topic to their personal experience—topic domination. The thread ends as Poster_C repeatedly posted about their personal experience to control the topic and caused abrupt topic drift.

Poster_A: *I have heard that minutes makes a difference concerning a stroke, could seven hours make a difference with a blood clot beginining in the uppper leg traveling down?*

Poster_B: *I don’t know how long it takes for tissue to die, but I would not wait 7 hours. But more important the clot can breakup and go to the lungs.*

Poster_C: *My mother died waiting for 7 hours, before she was taken to hospital. She was refused transport by ambulance service, because of misdiagnosed by Paramedic.*

MD_Poster_D: *It could - the longer tissues are deprived of blood and oxygen, the greater the risk of having permanent damage. Always better to seek medical attention earlier when there are concerns of a stroke or of other similar types of issues.*

Poster_C: *Thanks Dr. [Name], I feel she could have been saved, if she had gotten treatment sooner. The doctors will not say one way or the other, they afraid of being ask to testify in court.*

MD_Poster_D: *I’m so sorry to hear about your loss – it’s really helpful for other people in this forum to hear about your experiences - so thank you for sharing them with us.*

Poster_C: *Dr. [Name], Thanks for your welcome response. You seem like a caring and knowledgeable Doctor. I would like to talk to you further about this situation, My email address is [email address]* (Example Thread 1 from Heart Disease community)

We observed that sharing personal experience pertaining to the main thread topic was commonly practiced. Although personal narratives can provide powerful information [27], they can also prompt topic drift when shared in the middle of threads as shown in Example Thread 1 above.

Another source of abrupt topic drift was requests to MD moderators. Many community members asked MDs personal questions in the middle of the threads, similar to Poster_C in Example Thread 1 above. Other causes of abrupt topic drift included jokes or the inability of community members to use the online interface. For example, members started new conversations or sent personal messages from within the thread, then excused themselves for changing the topic:

“Hi guys, it maybe kinda off topic. I actually don’t know how to post my own topic (I’m new here, sorry.) […]”.

Sexual Health Community

**Table 3:** Usages of the key terms.

	Local only	Global only	Both	Total
Hijack	34	0	7	41
Off topic	33	23	21	77

Abrupt topic drift occurred from multiple sources, including members’ desire to joke, share personal stories, or interact with MD members as well their inability to use the interface. Although complete prevention of abrupt topic drift may not be possible, some can be addressed through better design (see Discussion).

### RQ2. What Are the Reactions and Meta-Discussions on Topic Drift in Explicitly Identified Topic Drift Threads?

We found 185 posts from 168 unique threads: 53 posts in which community members used the key term “hijack” and 132 posts in which community members used the key term “off topic”. We also found 2894 posts from 373 threads that contained either key term in the title. However, we did exclude the latter from analysis. Both members and moderators used the terms. After applying these criteria, only 118 posts from 114 unique threads were considered in this analysis.

“Hijack” was associated with local topic drift whereas “off topic” was used to indicate both local and global topic drift. The types of topic drift were not mutually exclusive (Table 3).

Two major themes emerged from qualitative analysis and are presented below. First, we found evidence of a posting culture in members’ reactions towards abrupt topic drift (ie, hijacking and off-topic discussions). Second, contrary to previous research, we found that members supported having off-topic discussions (ie, global topic drift).

#### Posting Culture With Respect to Abrupt Topic Drift

The following was a canonical example of how a community member believes threads should start and unfold in WebMD communities.

It is usually best to start your own discussion if you have questions or are seeking support. Certainly, you can share your own experiences and that is encouraged here. […] Elaborating too much is sometimes considered “hijacking a thread” in internet message board lingo. Many times this happens in these discussions - they take many tangents with different twists and turns. […] Regardless of how a discussion evolves, I always pray that we all can find the answers and relief we need.

Pain Management community

As shown in the example post above, the community member was aware of topic drift and described it with the term “hijacking.” According to the member, hijacking could occur when a member elaborates too much or otherwise dominates a thread. Dominating the conversation has been associated with topic control and topic drift in previous studies [7,21,29] because the dominant participant frequently changes the current topic to their own areas of interest. In the last sentence of the example post above, the member indicated how topic drift could affect the original poster in obtaining desired help. Furthermore, the member showed an intuitive understanding that the main purpose of a thread is to answer or give support to the original poster. To illustrate, the original posters shows frustration when the topic drifts:

“Why do my post always get treated as if I am posting something none [no one, sic] needs to know I do not think I will post here anymore, :angry: [name]”.

Diabetes community

According to Lambiase [7], off-topic discussions are associated with discontinuation or inactivity by community members. Similarly, we observed frustration of original posters when the topic drifted in the middle of threads as shown in the example above. Furthermore, we found apologetic behavior shown by community members who caused the topic to drift. The following example post is a response to the example post above in which the member apologizes to the original poster for changing the topic after being confronted:

“I am sorry I hijacked your thread, [name]. That is a bad habit of mine. Your post IS valuable. […] Truly, [name], I didn’t mean to hurt your feelings. I am sorry.”

Diabetes community

Because WebMD members showed an intuitive understanding of the thread’s main goals, members worked to counteract topic drift by bringing the conversation back to the original purpose of the thread as shown in the following example post:

“Since your question seems to have gotten hijacked by a debate about the economy and the merits of various forms of education, I’ll try to get it back on track […]”.

Sexual Health community

Moreover, experienced community members knew the sensitivity of certain topics, such as religion, that could easily become the main topics of the conversation through *chained explanation* (ie, explanation that seems more interesting than the current topic and becomes the new topic) [1]:

“As for the Christian aspect, I hesitate to go there at all because in my observation of past threads, this tends to hijack the main topic completely […]”.

Sexual Health community

The posts above show how community members reacted negatively to local topic drift and its negative effect on the main topic. These examples of topic drift often occurred in the middle of threads as conversations evolved. In contrast, members described starting off-topic discussions with regard to goals of the specific community (ie, global topic drift) positively, which we describe next.

#### Useful Purposes of Sharing Off-Topic Discussions

Although members reacted negatively towards local topic drift in the middle of the thread, they reacted positively to off-topic stories (ie, global topic drift) when shared separately in new threads. The following posts show the reaction of moderator and user member towards global topic drift.

To all re your comments about staying on point....if this community were being taken over with off-topic and/or “fun” discussions, that would be one thing. But that’s not the case in this community or even on this thread. Yes, on any board there are newcomers and lurkers. They get good information and support here. But, to me, a bit of fun can also add to creating a community where someone would like to stay for a while.

Diabetes community moderator

Personally, if all that was discussed in any community on WebMD was the main topic, I would cease to be involved. I enjoy sharing with others and getting to know them by discussing what is happening in their lives other than the main health concern.

Diabetes community member

Members showed support for having off-topic discussion because it could build rapport and bring members closer. However, members also suggested ways to indicate that the topic of the thread was unrelated to the health condition of the specific community. For example, adding either *OT* or *off topic* in the title was suggested or practiced in 5 communities (ie, ADHD, Breast Cancer, Diabetes, MS, and Sexual Health) as shown in the quote: “‘OT’ means ‘off topic.’

It lets people know the subject won’t be MS. Otherwise, someone will click on it expecting to find MS info, then they may get upset when they find that it’s not what they wanted.”

MS community

Moreover, we found that off-topic threads were significantly longer (mean 7.76 posts) than on-topic threads, with an average 6.76 posts (U=5456222, *P*=2.69e-05, *r*=.13). Our findings suggest that members reacted negatively towards local abrupt topic drift and topic control similar to previous studies [7,21,29]. However, we extend the literature by identifying novel benefits of global topic drift in online health communities.

### RQ3a. Manual Analysis: Who Counteracts Topic Drift?

For the first phase, we examined counteraction to topic drift through manual analysis of 70 threads, including 416 posts from 50 threads used in RQ1 with an additional 187 posts from 20 new threads. We found counteraction in 13 of the 70 threads (19%). Of the 13 threads with counteraction, 6 were made by original posters, 5 were made by other users, and MDs and moderators made the remaining 2 counteractions to topic drift. Next, we present qualitative themes that emerged from our analysis of counteracting topic drift.

#### Original Posters Put the Most Effort Into Counteracting

Threads with highly active original posters tended to stay on topic better than threads with fewer active original posters. Original posters reposted to their own threads in 37 out of 70 threads (53%). Below is an example thread (Example Thread 2) from the Heart Disease community in which the original poster provided counteraction to topic drift:

Poster_A: *My roommate is not yet 40 and has had to have 3 stints in the last year. Now the Cardiaologists are saying that he needs a pacemaker and most likely was born with Bradycardia. What exactly is Bradycardia and are we looking at a not so good prognosis for his future? Isn’t he somewhat young to be needing a pacemaker and what if the pacemaker does not have the expected result? What is the next step?*

Poster_B: *Bradycardia just means a heart rate of less than 60. That in itself is not a problem. The problem is when it is not beat fast enough to keep up with demand. Here is some information on the causes and treatment.* [URLs]

MD_Poster_C: *Bradycardia means a low heart rate, usually less than 60 beats per minute. A pacemaker can be recommended when bradycardia is symptomatic, or if there is another underlying electrical problem with the heart that increases the risk of the heart slowing even more or even stopping. Pacemakers work very well […]*

Poster_D: *Dear Dr. [Name], My mother is 73 years old, and had a pacemaker placed 2 years ago at the, Mayo Clinic. She is doctoring in her home town now. They are having trouble contoling her comidon levels, it has been 2 weeks now, and still do not have the levels controled. Is this unusual to have it take so long to adjust her levels?*

Poster_A: *Thanks for your reply. One more question. How does all of this associate with the stints and I forgot to mention that my friend has had two heart attacks this past year. Can we possibly look forward to my friend having a long and somewhat healthy life if the pacemaker and his new medication, Coreg, do what they are supposed to do? I realize that I am asking you to look into a crystal ball, but surely you have an educated guess?* (Example Thread 2 from Heart Disease community)

In Example Thread 2, Poster_A is the original poster who started the thread with multiple questions including (1) bradycardia and (2) possible outcomes and expectations. Both Poster_B and MD_Poster_C focused on bradycardia and treatment options (eg, uniform resource locators [URLs] and pacemakers). Poster_D, however, changed the topic to Poster_D’s personal question and attempted to engage in a side discussion with the MD_Poster_C. The original poster, Poster_A, counteracted this drift by bringing the topic back to the unanswered question by elaborating on their situation. The 2 most common ways original posters counteracted topic drift were (1) focusing the discussion to the remaining unaddressed issues and (2) correcting the discussion trajectory (eg, “this is about X not about Y”).

In our manual analysis, we found that original posters put in the most counteraction effort. Other users and members with defined roles (ie, MD and staff moderators) also counteracted topic drift. However, they also went along with the current topic of conversation at times. Similar to original posters, MDs, moderators, and other users counteracted topic drift by (1) addressing unanswered questions after topic drift had occurred or (2) discouraging abrupt topic drift (eg, *“I urge you to start another discussion”*).

**Table 4:** Mean counteraction effort, standard deviation, and 95% confidence interval for different roles of members.

Role	Counteraction effort (SD)	95% CI
Original posters (n=6233)	0.61 (0.42)	0.60 to 0.62
Staff/MD (n=33)	0.46 (0.26)	0.36 to 0.55
Power user (n=94)	0.53 (0.13)	0.50 to 0.56
Regular user (n=33,469)	0.35 (0.46)	0.34 to 0.35

**Table 5:** A pairwise comparison of counteraction by role.

First role	Second role	U	Difference of means (Second – First)	Adjusted *P* value	95% CI	*r*
Original posters	Power user	358,110	-0.08	<.001	0.11 to 0.28	.22
Staff/MD	Original posters	75,350.5	0.15	.01	-0.33 to -0.04	.27
Staff/MD	Power user	1194	0.07	.05	-0.16 to 3.35e-06	.23
Power user	Regular user	227,792	-0.18	<.001	0.36 to 0.47	.86
Regular user	Original posters	1,087,761	0.26	<.001	-0.14 to -3.68e-05	.99
Regular user	Staff/MD	412,935	0.11	.01	-0.33 to -0.04	.25

**Table 6:** Confusion matrix of automated topic drift detection technique.

		Gold standard	
		Positive	Negative
Similarity score	Positive	53	23
	Negative	21	50

### RQ3b. Automatic Analysis: Who Counteracts Topic Drift?

For the second phase, we automatically measured who counteracts topic drift most, using cosine similarity. Table 4 summarizes counteraction effort for each community member role. In total, 6233 original posters reposted to threads they initiated. On average, those original posters counteracted topic drift 61% of the time. Their effort to stay on topic exceeded that of any other role, similar to our finding in the qualitative analysis of RQ3a.

When we compared counteraction effort among roles, we found a significant difference (X^2^_3_=1715.70, *P*<.001). Table 5 shows post-hoc comparisons between specific roles. Original posters provided significantly more counteraction than other roles. In contrast, regular users provided significantly less counteraction effort compared to other roles. The effect sizes between power users and regular users as well as between regular users and original posters were considered large (>.50), while the effect sizes were small (<.30) for the other 4 pairwise comparisons. Findings indicate that original posters contribute most to counteraction effort and that this effect is large compared with regular users.

### RQ4. How Accurately Can Topic Drift Be Detected Automatically?

**Figure 1:**
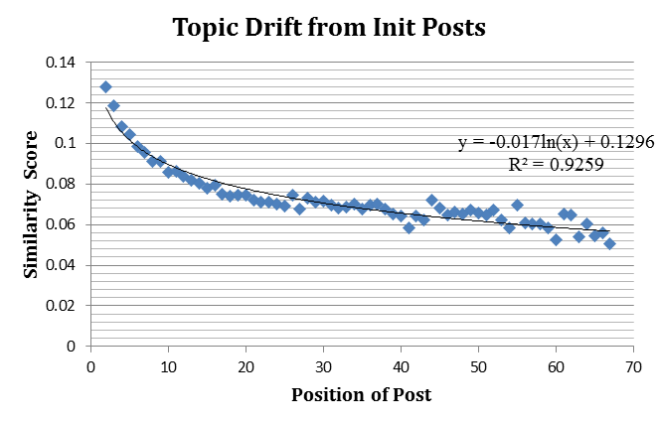
The general trend of topic drift in seven WebMD communities.

We automatically measured local topic drift using a cosine similarity. Figure 1 shows topic drift as threads evolved across all 7 communities. The x-axis indicates position of the posts in threads, and the y-axis indicates average similarity scores for posts in that position compared with the original post across the 7 communities. We captured the average similarity scores for positions with 50 or more posts. We applied logarithmic regression (y=-0.017ln(x) +0.1296), which resulted in a relatively high r-squared value of .93. Individual WebMD communities showed a similar trend in which the topic gradually drifted as conversation progressed. Thus, our automatic measurement of topic drift showed a pattern of gradual topic drift in which some topics carried to the next posts. This pattern aligns well with our manual analysis in RQ1 as well as findings from an existing manually assessed topic drift study [7].

Next, we evaluated our automated technique for detecting topic drift. Our evaluation against the gold standard (ie, 74 positive cases and 73 negative cases) showed promising results as an application to track topic drift. Table 6 shows that the automated topic drift detection technique correctly predicted 53 out of 74 cases of topic drift and 50 out of 73 “on-topic” cases with little or no topic drift. Automatically detecting topic drift through similarity measurement achieved a precision of .70, recall of .72, accuracy of .70, and F1 score of .71.

### RQ5. How Accurately Can Topic Drift Counteraction Efforts Be Detected Automatically?

Next, we evaluated our automated technique for detecting counteraction to topic drift using our manual assessment of 50 posts as the gold standard. Table 7 shows results from blinded evaluation on 50 cases of automated classification against our manual judgement. The automated technique correctly predicted 18 out of 24 cases of counteraction and 19 out of 26 cases of topic drift, which achieved a precision of .72, recall of .75, accuracy of .74, and F1 score of .73.

**Table 7:** Confusion matrix of automatically detecting counteraction to topic drift.

		Gold standard	
		Counteraction	Topic drift
Similarity score	Counteraction	18	7
	Topic drift	6	19

## Discussion

### Principal Findings on Topic Drift in Online Health Communities

Our findings shed light on how topic drift unfolds in online health communities, how members of these communities react to topic drift, and who brings topics back to the original intent of threads through counteraction. We also address gaps in previous literature by illustrating possible benefits of having off-topic discussions, highlighting counteraction provided by different types of community members, and applying an automated method to detect topic changes at the thread level.

Topic drift occurred in our online community data at 2 levels: global (ie, community-level) and local (ie, thread-level). Previous studies associated topic drift from global goals with incoherence [3] and described enforcing conversational participants to stay on global topics as difficult [44]. Moreover, off-topic discussion at a global level can lead members to unsubscribe or remain inactive [7].

However, in the online health communities we analyzed, we found topics generally stayed within the global level (ie, topics related to the intent of a specific community) with the exception of *OT* or *off topic* titled threads that purposely discussed off-topic issues. Both power users and moderators supported these off-topic discussions representing global topic drift. The off-topic discussion supporters, however, advocated that the off-topic nature of threads be indicated in the title so that the threads would not interfere with other discussions that pertained to the global goals of the community. The supporters voiced the opinion that off-topic discussions could build rapport and bring members closer. Such support might not be representative of reactions towards topic drift more generally because we focused only on self-identified topic drift threads.

We found that having off-topic discussions, as indicated by global topic drift, positively affected online health communities. For instance, many off-topic discussions were lively and humorous, which was in direct contrast to the melancholy and serious tone of many on-topic discussions. Moreover, off-topic discussion threads (ie, threads with OT in the title) had higher levels of participation. However, we did not find evidence that regular users supported off-topic discussions in our manual assessment. We suspect that either our sample size was not large enough or that only experienced members (eg, high level of active participation or defined community roles) were aware of the culture of sharing off-topic discussions. We reached this conclusion because we observed posts that asked about the meaning of *OT* in the title. Given this confusion, we suggest that designers and administrators of online health communities consider other structured ways to have off-topic discussions (see further discussion below).

Although power users and moderators supported off-topic discussions at the global level, most members reacted negatively towards abrupt local topic drift. We observed 2 types of local topic drift: gradual topic drift and abrupt topic drift. Gradual topic drift, in which only a fraction of topics changed through a semantic parallel, occurred most frequently. This change is common and expected in any conversation [2] including CMC [7]. Members typically seemed to tolerate such gradual topic drift. However, members reacted negatively towards abrupt topic drift—when previous topics were completely replaced with different topics. When abrupt topic drift occurred, original posters showed frustration, and some community members even attempted to revert the topic back to the original topic. Although complete elimination of abrupt topic drifts could be difficult, some abrupt topic drift is likely preventable with improved design.

### Practical Implication for Online Community Use, Research, and Design

Many online communities use moderators and even community members to regulate the content of posts. Manual efforts of monitoring posts have been shown to miss or misjudge important posts [45]. Our automated method could be utilized to expand these efforts. For example, an automated method could be used to alert community members when topics of their posts are entirely different from the topic of the thread. Raising self-awareness could help to control topic drift.

Moreover, moderators could use automated methods as a supplement to reduce the burden of keeping discussions on track. Automated methods could alert moderators of abrupt topic drift occurring in the middle of threads. An immediate alert could allow moderators to provide timely support and minimize negative impacts. As for the community, similar automated methods could provide the basis for filtering spam or abusive content, while keeping relevant on-topic content available to the community.

Expanding these topic-oriented automated methods could further enhance online health communities by (1) locating topically relevant posts [46] in threads even if topic drift occurs and (2) identifying peers with shared circumstances [47]. Locating relevant information in large volumes of text can be daunting. An automated method could automatically locate previously written posts on a similar topic without delay. Moreover, such a system could provide opportunities to connect with members who previously discussed topics that reflect similar interests and experiences. Studies have consistently shown that patients find peer support more helpful when provided by fellow patients with similar experiences [48,49].

We also offer design considerations based on our findings. Our findings suggest that facilitating off-topic discussions could benefit members who desire emotional connection and lighten the mood of the community. The popularity of off-topic discussion threads also suggests that support for limited off-topic discussions could contribute to sustained participation, which is a prominent challenge for online communities [50,51].

We discovered that some members expressed a reluctance to change topics completely but did so anyway because starting a new thread or sending a private message was not an intuitive process. An intuitive interface supporting the creation of new topics or branching off a new side conversation might reduce abrupt topic drift.

### Limitation and Future Directions

One limitation in our qualitative analyses was that we had a single analyst and a dataset with 7 communities within the WebMD platform. Therefore results may not be generalizable to other communities. We recognize the limitation of using a single analyst. However, a previous study illustrated that the interpretation of topic drift can be subjective [11], thus we employed a systematic approach. We acknowledge that our large sample size could have inflated the statistical significance levels and raises questions about the practical significance of our quantitative findings. We completed effect size estimates to aid our interpretation of results.

We also considered observations within the unit of analysis (ie, role within a thread) as independent; nevertheless, correlation could exist in the counteraction effort a member provides when acting in different roles. However, both qualitative and quantitative analyses showed consistent results in a diverse group of online health communities in WebMD. Findings could indicate that original posters have a higher stake in keeping the thread on topic than other members. This finding, however, could also be due to differences in the responsibilities of moderators and other types of community members.

From previous research, we expect moderators to recruit new members, temper discussions, and create an engaging and respectful community culture [22]. Although we are uncertain of the specific obligations of WebMD moderators and MDs, it is reasonable to assume that they attend to many threads to create an engaging and respectful community culture. Due to their demanding responsibilities, moderators and MDs could miss topic drift in threads. Conversely, original posters might be more invested in their own threads, thus providing substantial effort to keep thread topics aligned with their interests to obtain desired support. Future work using mixed methods, such as surveys and interviews, could ask original posters about effects of topic drift or ask about responsibilities of the moderators to gain a deeper understanding.

Our findings suggest that topic drift occurs despite apparent differences in health aspects (ie, biological, psychological, and sociological) and representative demographics (ie, age and gender) of different communities. Understanding how these differences affect topic drift could deepen our understanding in future work. Although our term-based similarity metric was not developed to analyze conversations, our study showed its practical application for analyzing CMC through consistent results across the seven diverse WebMD communities. An extended evaluation using a large gold-standard dataset could investigate the effectiveness of this as well as other sophisticated similarity measurements, such as knowledge-based [52] and corpus-based [53] approaches to automated detection of topic drift. These sophisticated similarity measurements that consider semantic meaning or syntactic organizations of the words could improve the performance of topic drift and members’ counteraction detection.

### Conclusion

We provide new insights into topic drift by illustrating possible benefits of having global topic drift in online health communities, identifying sources of abrupt local topic drift, highlighting considerable counteraction provided by original posters, and creating automated methods to detect topic drift and counteraction at the thread level. Our findings suggest that members react negatively towards local topic drift in the middle of the thread but advocate sharing globally off-topic stories to build rapport and bring members closer. Although many members counteract topic drift, original posters appear to provide the most effort to keep their threads on topic. Finally, we demonstrated automated techniques to detect both topic drift and counteraction. Based on these findings, we have contributed practical suggestions for designing online health communities to better facilitate online discussions. Findings from this study have the potential to reduce topic drift and improve online health community members’ experience. Such experiences could improve the personal health management of members who seek essential information and support during times of difficulty.

## Abbreviations

CMC: computer-mediated communication
ADHD: attention deficit hyperactivity disorder
ADA: Americans with Disabilities Act
tf-idf: term frequency-inverse document frequency
MS: multiple sclerosis
MD: medical doctor
TL: thread length
OT: off topic
URL: uniform resource locator
